# The distribution and abundance of an unusual resource for koalas (*Phascolarctos cinereus*) in a sodium-poor environment

**DOI:** 10.1371/journal.pone.0234515

**Published:** 2020-06-11

**Authors:** Sarah Martin, Kara N. Youngentob, Robert G. Clark, William J. Foley, Karen J. Marsh

**Affiliations:** 1 Research School of Biology, The Australian National University, Canberra, Australia; 2 Research School of Finance, Actuarial Studies and Statistics, The Australian National University, Canberra, Australia; University of Sydney, AUSTRALIA

## Abstract

Environmentally available sodium tends to decrease with increasing elevation, and sodium resources in these sodium-poor environments are critical for the survival of herbivores. Eucalypt leaves in the subalpine Monaro region of NSW, Australia contain much less sodium than eucalypt leaves at lower elevations, and subalpine koalas obtain this much needed resource by eating the bark from some *Eucalyptus mannifera* trees. To better understand the availability of salty-barked trees, we searched for evidence of koala bark chewing at 100 randomly generated locations in the region. We found 318 *E*. *mannifera* trees with koala chew marks. We also analysed sodium concentrations in the bark of three unchewed *E*. *mannifera* trees from each site to determine whether there were trees with high bark sodium content that had not yet been utilized by koalas. Although 90% of unchewed trees had sodium concentrations less than 225.4 mg.kg^-1^ DM, some unchewed trees contained high sodium concentrations (up to 1213.1 mg.kg^-1^ DM). From the random survey, we can extrapolate that 11% of trees in this area have bark sodium above 300 mg.kg^-1^ DM, which is based on the concentration of bark sodium observed in at least moderately chewed trees. We would expect to find 0.24 of these trees per 200 m^2^, or 720,000 salty-barked trees in the 30 km by 20 km study area. Bark chewing by koalas is widespread in the area, and trees with salty bark are more common than initially thought. We discuss correlations with the occurrence of salty-barked trees and other landscape attributes; however, questions remain about why some *E*. *mannifera* trees have much more bark sodium than others. Studies such as this one should be expanded to identify sodium resources and their availability for other herbivorous species, since many are predicted to move to higher elevations in response to climate change.

## Introduction

Sodium is critical to the survival of all mammals. It is required for many essential bodily functions, from osmotic homeostasis and nerve transmission, to reproduction and lactation [1, 2]. However, sodium is typically less available in plant-based diets than meat-based diets [3]. As a result, herbivores may consume dirt, bark, and other substances that are not typical of their usual food in an attempt to obtain this important nutrient [1, 4, 5, 6].

The hunger for salt is even more pronounced in herbivore populations at higher elevations, since environmentally available sodium tends to decrease with increasing elevation [2, 4, 7]. Sodium can be toxic to plants, so most plants do not accumulate it, making sodium easily leached from ecosystems [8]. Sodium loss from upland areas is exacerbated by freeze-thaw cycles, which break up the soil, combined with melting snow that leaches out any soluble sodium present [4]. The transport and deposition of salt via aerosols also declines with distance from the ocean [9, 10]. Therefore, non-foliar sodium resources at higher elevations are likely to be critical for maintaining populations of herbivores that are unable to meet their sodium requirements by only eating leaves.

Despite its importance to the survival of animals, sodium and its availability can be overlooked in wildlife management and conservation, particularly for specialist folivores that are thought to meet the bulk of their nutritional requirements from tree leaves. Recently, Au et al. [7] found that koalas (*Phascolarctos cinereus*) living in the Monaro region of New South Wales, Australia engaged in an unusual bark-chewing behaviour to obtain sodium in an otherwise sodium-poor environment. The Monaro region encompasses some of the highest-elevation sites at which koalas occur (900–1,300 m). Leaves from *Eucalyptus* species in the area were found to be low in sodium compared to eucalypt leaves sampled at lower elevations, and koalas in this region are unlikely to meet their sodium requirements from *Eucalyptus* leaves alone [7]. The bark from chewed *Eucalyptus mannifera* trees was substantially saltier than nearby unchewed trees, regardless of eucalypt species. At the time, only 34 chewed trees, all *E*. *mannifera*, had been identified in the region.

Given the potential importance of this sodium resource for maintaining koala populations in an otherwise sodium-depauperate landscape, we conducted a large-scale survey to estimate the abundance of *E*. *mannifera* trees with high concentrations of bark sodium across the study area. This information is essential to determine whether additional conservation measures should be afforded to salty-barked trees to help maintain koala populations living in this environment. The reason that the bark of some trees is salty while others are not is unknown, so we also investigated potential relationships between salty-barked trees and other environmental factors that might provide insights into the underlying mechanism responsible for the occurrence and distribution of these trees in the landscape. Studies such as this one should be expanded to identify sodium sources and investigate their availability for other herbivorous species in sodium-poor environments, particularly since many animal populations are projected to move to higher elevations in response to anthropogenic climate change [11].

## Materials and methods

### Study area

The study area was in the Monaro Region of New South Wales, Australia, which is a plateau of grassy woodlands that range in elevation from 750 m to 1300 m above sea level (central coordinates: -36°02’11.92 S, 149°18’19.68 E). Large-scale tree clearing over the past century has converted much of the native woodland to pasture, but remnants and large patches of native woodland and dry sclerophyll forest remain. The dominant tree species include *E*. *mannifera*, *E*. *dives*, *E*. *rossii*, and *E*. *macrorhyncha* with an understory of *Acacia spp*. The study area encompassed private and state forest land tenures, and landholder permission to access properties was sought at the outset and throughout the duration of the project, with assistance from the NSW Office of Environment and Heritage (OEH).

Yellow and grey-brown podsolic soils are characteristic of the region, and they are typically poorly aerated and low in fertility [12, 13]. The underlying geology is comprised of Palaeozoic Era sediments that metamorphosed during the creation of the Lachlan Fold Belt. Fluvial sandstones and lacustrine sediments are interspersed and interbedded with granite plutons and alkali basalts from ancient volcanic activity [14]. In addition, the area has thick calcrete profiles (regolith containing calcium carbonate) that are indicative of low rainfall as a result of the rain shadow from the Snowy Mountains to the west. The average annual rainfall is about 550 mm, with a pronounced peak in summer (December to February—Australian Bureau of Meteorology, 2016). The temperatures in summer average 9°C to 27°C, and −3°C to 13°C in winter (mean max and mean min—[15]).

### Pilot study

During autumn and winter 2017, we resampled 20 chewed trees and their unchewed neighbours (total n = 40) that had previously been sampled in spring 2015 by Au et al. [7]. This was to confirm that the differences detected had been maintained over years, and that the observations were not season-specific. Samples were collected and analysed following the methods described in Au et al. [7], which are also described in more detail in the relevant sections below.

### Selection of sites for regional tree survey

**Fig 1:**
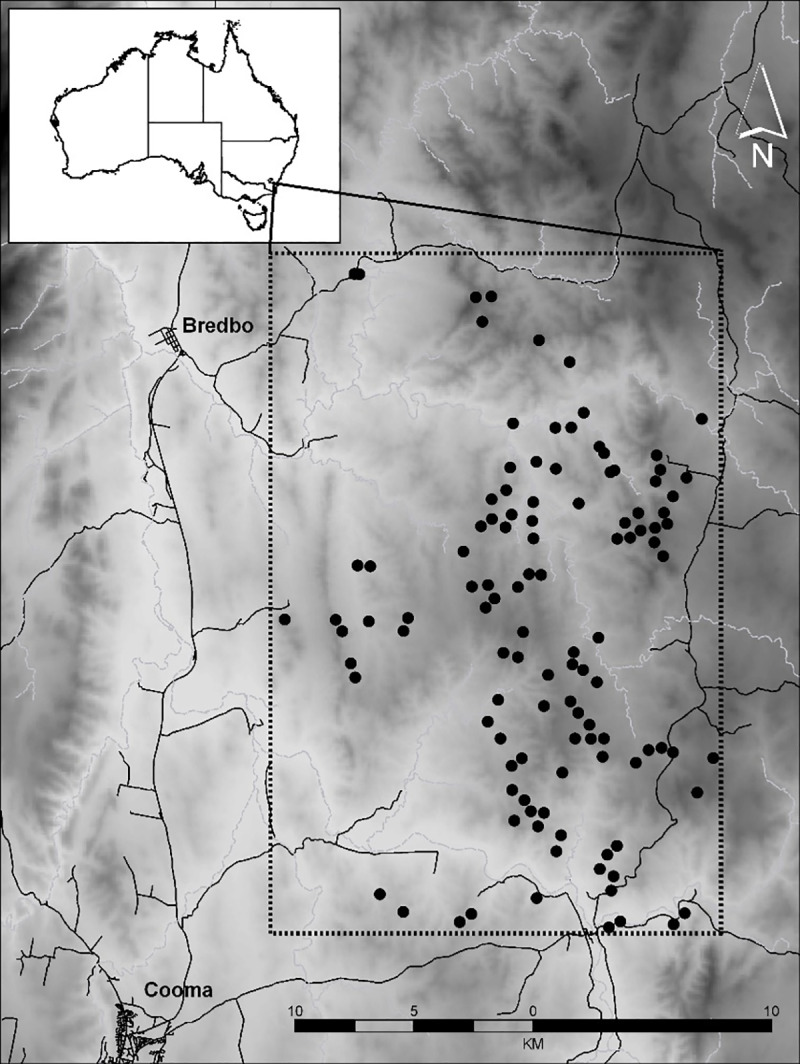
**Map showing the designated study area (black rectangle) and the 100 surveyed sites (black circles), with the towns of Bredbo and Cooma marked as reference points.** Roads are represented by solid black lines and waterways by thin grey lines. Elevation is represented on a shading scale from white (750 m) to dark grey (1250 m).

In consultation with the NSW OEH, we designated a study area perimeter (approx. 20 km x 30 km) that encompassed the area in which chewed trees had been observed [7], as well as a larger surrounding area of conservation interest (Fig 1). The Excel command ‘RANDBETWEEN’ was used to randomly generate eastings and northings coordinates for 100 sites within the defined study area (Fig 1). If randomly chosen coordinates fell within paddocks without trees, within 500 m of known chewed trees (to avoid pseudo-replication), within 500 m of another generated coordinate (to avoid overlap), or on the properties of landholders that had not granted access permission, they were discarded and a new random coordinate selected.

### Survey for chew marks and collection of bark samples

The one hundred selected sites were visited between June and August 2017. At each site, one unchewed *E*. *mannifera* tree was designated the “focal tree”. If there were no *E*. *mannifera* trees within approximately 50 m of the site coordinate (n = 13 sites), the lack of *E*. *mannifera* was recorded, and the nearest *E*. *mannifera* was designated as a focal tree. All *E*. *mannifera* trees within a 50 m radius of the focal tree were counted and checked for chew marks from koalas. Trees with chew marks were assigned a degree of chewing (i.e. test: less than 2 cm^2^ chewed; light: 2–10 cm^2^ chewed; moderate: 10–20 cm^2^ chewed; heavy: 20–50 cm^2^ chewed; and extreme: 50 cm^2^ or more chewed). The age of the chew marks was classified into four categories, from very fresh to very old, following the descriptions provided by Au et al. [7]. Other site elements were also recorded, including the elevation, forest connectivity, the dominant *Eucalyptus* species, and whether undergrowth was dense or open. Tree species composition and density at each site was estimated by counting all *Eucalyptus* trees along a 50 x 2 m transect. Data was also obtained from Geoscience Australia, NSW Digital Dataset, for information about the sedimentary rock from which the soil was derived at each site [16].

Bark samples were collected under permit from the New South Wales Government (SL101934). We sampled all focal trees and their two closest *E*. *mannifera* neighbours (i.e. 300 trees in total) using a wood planer to scrape approximately 20 g of bark from the outer 1–3 mm of the trunk. This is similar to the depth of koala chew marks [7]. We recorded the latitude, longitude, elevation, diameter at breast height (DBH), and the direction of the slope, if applicable. Bark samples were dried in an oven at 70°C for at least 48 h, and then ground in a Tecator Cyclotec 1093 sample mill (Foss, Hillerød, Denmark) until they passed through a 0.5 mm mesh.

### Analysis of sodium in bark

Sodium concentrations in bark were analysed using inductively coupled plasma atomic emission spectrometry (ICP-AES). One hundred samples (one per site) were prepared using a microwave digestion protocol, in which 0.180–0.220 g dried, ground sample was digested in 8 mL 70% nitric acid and 2 mL 36% hydrochloric acid in a Milestone—Start D—Microwave Digestion System (Milestone Inc., Shelton CT) using US EPA Method 3051. These samples were subsequently analysed on a Varian Vista-Pro Inductively Coupled Plasma-Optical Emission Spectrometer with the following conditions: power 1.20 kW, plasma flow 15.0 L.min^-1^, auxiliary flow 1.50 L.min^-1^, nebuliser flow 0.90 L.min^-1^ (SeaSpray Nebulizer with a high salts cyclonic spray chamber (both from Glass Expansion, Melbourne, Australia)), injector 2.4 mm capillary style (part of the fully demountable torch used, produced by Glass Expansion), argon humidifier used on injector gas. The sample introduction conditions were fast pump, sample delay/rinse, uptake delay 45 s, rinse time 40 s, stabilisation delay 15 s, caesium chloride ionisation suppression at 1000 ppm Cs (with scandium as an internal standard). Standards were prepared to encompass previously reported sodium values in bark (8–2000 mg.kg^-1^).

Due to issues in accessing this instrument, the remaining samples were digested using the following protocol. Dried, ground bark (200–205 mg) was weighed into acid washed Pyrex tubes, to which 5 mL HNO_3_ (69%) was added. The tubes were heated to 125°C for 30 min followed by 130°C for 2 h, using an Ai Scientific AIM500 Hot Block Digestion system controlled by an Ai Scientific AIM 500 programmable controller (Brisbane Australia). The tubes were cooled for 15 min, and then 2 mL HCl (37%) was added. Tubes were heated again (130°C for 30 min, followed by 140°C for 30 min, and finally 145°C) until the volume of acid had reduced to 200–300 μL. Once the tubes had cooled completely, the contents were mixed on a vortex mixer and made up volumetrically with Milli-Q water to 10 mL. Samples were left to settle for 24 h, and were then decanted into 15 mL Falcon tubes.

Samples were analysed on a VISTA AX CCD Simultaneous ICP-AES. The commercial standard used was AccuTrace^TM^ Reference Standard Sodium, Plasma Emission Standard (ICP), 1000 μg.mL^-1^ sodium nitrate, which was diluted as needed, to produce three standards of 0.1, 1 and 10 ppm sodium nitrate. The machine was rinsed with 2% HNO_3_ for 2 min between samples. Microwave digestion and block digestion provide comparable results for the analysis of sodium in plant material [17].

### Statistical analysis

#### Pilot study

A sample of 40 chewed and unchewed trees were selected and measured for bark and leaf sodium in 2015 by Au et al. [7]. These trees were revisited in 2017 and their sodium content re-measured. One tree sample was left out due to measurement error, leaving 39 samples. A paired t-test was used to compare the 2015 and 2017 bark sodium levels, and a two-sample t-test was used to compare the 2017 bark sodium levels for the chewed and unchewed trees.

#### The relationship between the number of chewed trees and other site attributes

We fitted beta-binomial multiple regression models using the gamlss package [18] in the R Statistical Environment [19] to determine whether the number of trees with koala chew marks at each site depended on any of the following environmental factors: whether the dominant *Eucalyptus* species at the site was in the *Eucalyptus* (common name, monocalypt) or *Symphomyrtus* (common name, symphyomyrtle) sub-genus, the logarithm of tree density, elevation, whether the forest was contiguous or patchy, the sedimentary rock from which the soil was derived (three categories), whether the site was flat or sloped, the easterly gradient of the slope (calculated as the sine of the approximate slope direction), the northerly gradient of the slope (the cosine of the approximate slope direction) and whether the understory was dense or open.

Two beta-binomial regression models were fitted, with dependent variables the count of trees with any degree of chewing, and the count of trees with moderate or more chewing. All of the above covariates and factors were included in each model, as the variance inflation factors were all below 1.3, rather than conducting a variable selection process, which would have added another source of uncertainty. Three sites were excluded from analyses as they had rare sedimentary rock (substrate) types containing only two or one sites, which left 97 sites for fitting of the regression models. The *p-*values for individual covariates and binary factors were calculated using Wald chi-square tests, and the *p-*value for the substrate type factor (which had three levels) came from a likelihood ratio chi-square test. Beta-binomial models were used because they allow for chewing activity to be correlated within sites, whereas a binomial model would have implied independence for every tree.

#### The relationship between the sodium concentration of unchewed trees and other site attributes

The environmental characteristics associated with bark sodium concentration were investigated using a linear mixed effects model, fitted using the lme4 package in R, with site as the random effect. The dependent variable, sodium concentration, was log-transformed to achieve closer to a normal distribution for this variable. The independent variables were the same as those in the preceding beta binomial models, except that tree DBH was included and elevation for individual trees was used rather than site elevation. Data were available for 285 trees (initially 3 trees in each of 100 sites, but 3 sites with unusual rock types were removed, and six sodium analyses were discarded because of measurement error).

#### Number of salty-barked trees in the area

Trees were considered to have salty bark if the concentration of sodium in the bark was 300 mg.kg^-1^ or higher. This was derived from the lowest 5^th^ percentile of bark sodium concentration found in trees with at least moderate levels of chewing based on earlier research [7]. Sodium concentration was measured in the bark of three *E*. *mannifera* trees per sampled site. The number of *E*. *mannifera* trees was also recorded for a 50 x 4 m strip transect at each site. The number of salty-barked *E*. *mannifera* trees in each of the 100 strip transects can therefore be estimated by the proportion of measured *E*. *mannifera* trees multiplied by the number of *E*. *mannifera* trees. The percentile bootstrap confidence intervals were calculated using the R package boot [20], resampling site-level estimates.

## Results

### Pilot study

The sodium concentration in the 39 re-collected bark samples from Au et al’s [7] study (re-collected for in this study in March, 2017) was lower than the values from their initial collection in September-October, 2015 (t_38_ = -3.22, *p* = 0.003). However, the sodium concentration in the bark of chewed trees (mean ± SD, 461.2 ± 407.8 mg.kg^-1^) remained significantly higher than unchewed trees (141.2 ± 213.7 mg.kg^-1^, t = 3.13, *p* = 0.003). This confirmed that differences in bark sodium concentrations between trees could still be detected, and that it was appropriate to undertake the larger survey of sodium concentration in the bark of *E*. *mannifera* across the study area during the winter of 2017.

### How common are chewed *E*. *mannifera* trees in the study area?

Out of the 5730 *E*. *mannifera* trees surveyed across 100 sites, 318 (5.55%) had chew marks from koalas. While walking to the sites, we also found an additional 36 *E*. *mannifera* trees with koala chew marks. The majority of the *E*. *mannifera* with chew marks had either test (33.8%) or light (33%) amounts of chewing. However, 19.9% of trees with chew marks had moderate amounts of chewing, while 8.8% had heavy chewing and 4.5% were extremely chewed. The majority of the chewed trees had old (28.1%) or very old (45.7%) chew marks, with only 26.1% of the chew marks being either very fresh or fresh. No other eucalypt species showed moderate or higher levels of chewing, although 18 *Eucalyptus rossii* had test or light chew marks. *E*. *rossii* trees were not included in statistical analyses.

### The relationship between the number of chewed trees and other site attributes

The number of chewed *E*. *mannifera* trees (with any degree of chewing) at a site was significantly associated with the dominant eucalypt subgenus and the easterly gradient (Table 1). The odds of a tree being chewed in a symphomyrtle-dominated site were 65% lower than in a monocalypt-dominated site (odds ratio = 0.350). Sites with some easterly slope also had higher odds of chewing, all else being equal. No other site variables significantly explained the number of chewed trees at the 5% level.

**Table 1:** Fitted beta-binomial regression model with dependent variable the number of chewed trees of any level of chewing at a site.

Site Attribute	Estimate	Std. Error	t value	*p-*value	Odds Ratio
Intercept	-5.108	1.316	-3.881	0.000	
substrate type				0.491	
igneous felsic intrusive	0.000				
metasedimentary siliciclastic	-0.320	0.433	-0.738	0.462	0.726
sedimentary siliciclastic	-0.452	0.365	-1.239	0.219	0.637
**Dominant Subgenus**					
** Monocalypt**	**0.000**				
** Symphomyrtle**	**-1.051**	**0.354**	**-2.971**	**0.004**	**0.350**
Flat	-0.587	0.405	-1.450	0.151	0.556
log(tree density)	0.070	0.223	0.315	0.754	1.073
patchy	0.342	0.537	0.637	0.526	1.407
northerly gradient	0.242	0.179	1.352	0.180	1.273
**easterly gradient**	**0.391**	**0.195**	**2.006**	**0.048**	**1.479**
elevation (‘00 m)	0.247	0.147	1.675	0.098	1.280

Results were similar for the model explaining the abundance of moderately to extremely chewed trees (Table 2). Symphomyrtle dominated sites had 57.4% lower odds of moderate or more chewing for any given tree (*p* = 0.032). Trees in higher elevation sites were also more likely to be moderately or more chewed: every 100 m in elevation was associated with a 63.3% increase in the odds of chewing (*p* = 0.007, odds ratio = 1.633).

**Table 2:** Fitted beta-binomial regression model with dependent variable the number of moderate or more chewed trees at a site.

Site Attribute	Estimate	Std. Error	t value	*p*-value	Odds Ratio
intercept	-8.145	1.589	-5.126	0.000	
substrate type				0.888	
igneous felsic intrusive	0.000				
metasedimentary siliciclastic	-0.181	0.577	-0.313	0.755	0.835
sedimentary siliciclastic	-0.242	0.493	-0.491	0.625	0.785
**Dominant Subgenus**					
** Monocalypt**	0.000				
** Symphomyrtle**	**-0.852**	**0.393**	**-2.170**	**0.033**	**0.426**
flat	-0.459	0.475	-0.966	0.337	0.632
log(tree density)	-0.120	0.267	-0.450	0.654	0.887
patchy	0.661	0.545	1.213	0.229	1.937
northerly gradient	0.147	0.219	0.671	0.504	1.158
easterly gradient	0.426	0.250	1.703	0.092	1.531
**elevation (‘00 m)**	**0.490**	**0.175**	**2.798**	**0.006**	**1.633**

**Fig 2:**
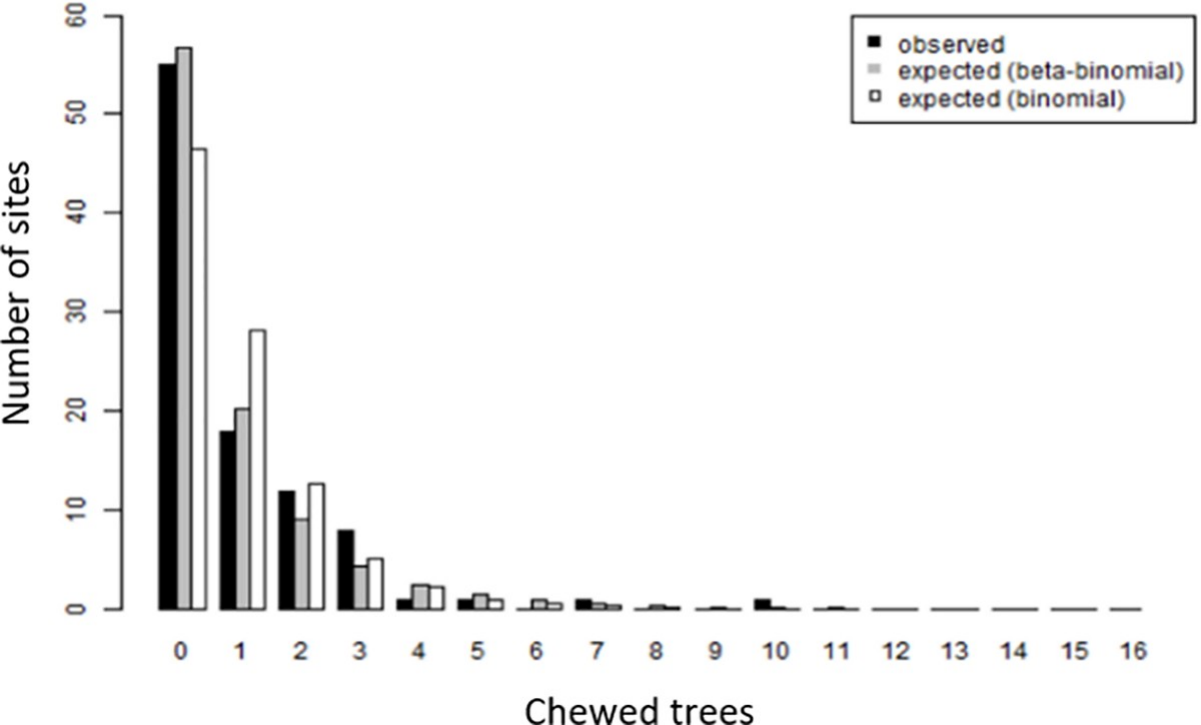
Observed distribution of the number of moderate or more chewed trees per site, compared to expected distribution under two models.

The beta-binomial models in Tables 1 and 2 had estimated sigma parameters of 0.038 and 0.015, respectively. These parameters reflect the degree to which chewing activity is clustered within an area. Because these parameters are difficult to interpret directly, and in order to visualise the degree of clustering, Fig 2 shows the observed distribution of the number of moderately chewed trees per site, as well as the expected distribution given the number of *E*. *mannifera* trees measured, for both the fitted beta-binomial model and for a binomial model, which assumes no clustering, with the same covariates. This shows that the observed spread of chewing activity varies more across site than the binomial model, indicating some clustering of chewing.

### What is the range of sodium concentrations in bark from unchewed *E*. *mannifera* trees?

**Fig 3:**
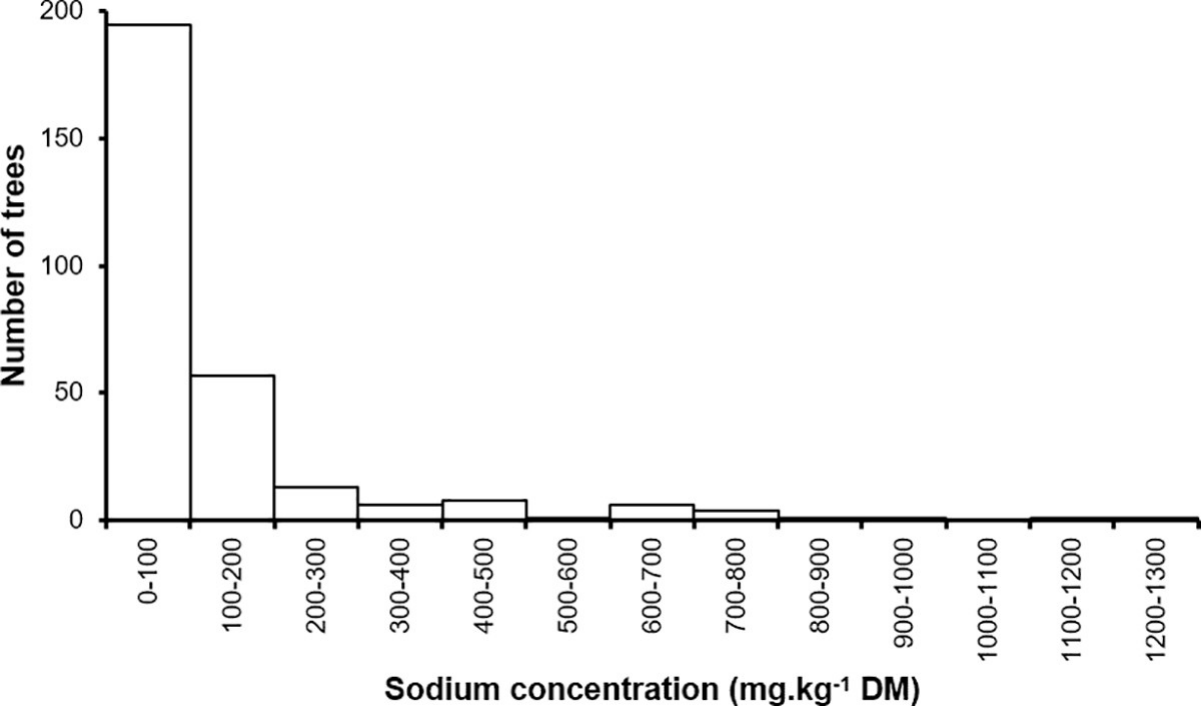
Distribution of sodium concentrations in the bark of 294 unchewed *E*. *mannifera* trees.

The range of sodium concentrations in bark samples from unchewed *E*. *mannifera* trees (n = 294) was 8.0 to 1213.1 mg.kg^-1^ DM (Fig 3). The bark from most unchewed *E*. *mannifera* had relatively low concentrations of sodium, with 90% of the samples containing less than 225.4 mg.kg^-1^ DM. The middle 50% of samples fell between 43.5 and 146 mg.kg^-1^ DM of sodium.

### The relationship between the sodium concentration of unchewed trees and site and tree characteristics

Flatness of the site was the only significant predictor at the 5% level (Table 3). Flat sites had sodium levels 85% higher than sloping sites, all else equal (t = 2.86, *p* = 0.005, exponentiated coefficient = 1.849). The estimated residual standard deviation was 0.89. The estimated standard deviation associated with sites was 0.44, indicating substantial clustering of sodium values within sites (*p* = 0.011).

**Table 3:** Linear mixed model of the logarithm of bark sodium content for 285 *E*. *mannifera* trees.

Site Attribute	Estimate	Std. Error	t value	p-value	Exponential of estimate
Intercept	4.780	0.827	5.781	0.000	
substrate type					
igneous felsic intrusive	0.000				
metasedimentary siliciclastic	-0.437	0.269	-1.624	0.108	0.646
sedimentary.siliciclastic	0.009	0.229	0.041	0.967	1.009
Dominant Subgenus					
Monocalyptus	0.000				
Symphomyrtle	0.233	0.170	1.371	0.174	1.263
**Flat**	**0.614**	**0.204**	**3.006**	**0.003**	**1.849**
log(tree density)	0.107	0.121	0.884	0.379	1.113
Patchy	0.356	0.330	1.079	0.284	1.427
northerly gradient	0.132	0.104	1.260	0.211	1.141
easterly gradient	0.000	0.124	0.002	0.999	1.000
elevation (‘00 m)	-0.069	0.085	-0.805	0.423	0.933
DBH (m)	-0.241	0.140	-1.714	0.088	0.786

### Number of salty-barked trees in the area

The total number of salty-barked *E*. *mannifera* in the wider region (which has area approximately 600 million m^2^) can be estimated by the total estimated salty barked *E*. *mannifera* in the 100 strip transects, multiplied by 600x10^6^ m^2^/(100 x 200 m^2^). The resulting estimate is that there are 720,000 salty bark *E*. *mannifera* trees in the region (95% confidence interval 310,000 to 1.25 million trees) which represents 11.1% of *E*. *mannifera* trees.

## Discussion

We found that there are more *E*. *mannifera* trees that are a potential sodium source for koalas in the study area than previously anticipated based on earlier work [7], and increased the number of known chewed trees from 34 to 352. The majority of unchewed *E*. *mannifera* trees had very low concentrations of sodium; however, some unchewed trees did have relatively high bark sodium concentrations (≥ 300 mg.kg^-1^ DM). Therefore, koalas have not yet exploited every salty-barked tree in the landscape. We found that both chewed trees and salty barked *E*. *mannifera* trees were spatially clustered, even after allowing for other site characteristics. Bark chewing marks were more likely to be found in monocalypt-dominated forests, on eastern slopes, and at higher elevation. Notably, this was not the case for salty barked trees that were unchewed. *Eucalyptus mannifera* trees were more likely to have higher bark sodium concentrations on flat, non-sloping areas. This information provides clues as to why only some *E*. *mannifera* trees have salty bark. However, future research is required to fully understand the mechanisms underlying the variability in *E*. *mannifera* bark sodium concentrations in this region.

The sodium concentrations that Au et al. [7] reported for the bark of chewed trees sampled in late winter/early spring (824.7 ± 435.4 mg.kg^-1^, mean ± SD) were higher than when the same trees were resampled in autumn for this study (461.1 ± 407.8 mg.kg^-1^, mean ± SD). Nevertheless, they were still significantly higher than their unchewed neighbours, indicating that salty-barked trees likely maintain high concentrations of sodium in their bark. Some studies have reported variation in sodium concentrations in plants across different seasons [21]. The decrease in the bark concentrations across sample periods suggests that the processes that lead to accumulation of sodium in the bark may be cyclical–perhaps over an annual cycle.

Excess sodium can be very damaging to plants, causing tissue damage, dehydration and reduced photosynthesis [22]. One of the ways that plants limit this damage is to transport excess sodium to the vacuoles of cells and export it into the phloem [23], where it can be deposited and shed in the bark [24]. In *Eucalyptus*, some species are more tolerant of saline conditions [25] and this is largely due to the accumulation of osmolytes in the leaves which prevent the cells from becoming dehydrated. These osmolytes (e.g., quercetin) are typically products of the flavonoid biosynthesis pathway which is notably rich in *Eucalyptus* [26]. As a feature of salt tolerance, some trees may be able to shed excess sodium more readily through their bark, and therefore have higher bark concentrations of sodium [23]. Bark shedding occurs in the spring and early summer coincident with the production of new leaf in *E*. *mannifera* [27]. Therefore, we might expect maximum concentrations in bark in the spring prior to bark shedding. These hypotheses could be tested with a longer time course of experiments and with the possible addition of sampling the phloem.

The finding that trees were more likely to be chewed at higher elevations is unsurprising given the relationship between decreasing foliar sodium concentrations in the environment and increasing elevation [2, 4, 7]. We know that foliar sodium in this area decreases with increasing elevations [4, 7], so it makes sense that koalas at higher elevation will be seeking out alternative sodium sources more often than koalas in lower elevations. The fact that chewed trees but not unchewed salty barked trees were more likely to be found in monocalypt-dominated forests than symphyomyrtle-dominated forests is difficult to decipher.

Koalas generally tend to prefer to forage from trees in the *Symphyomyrtus* subgenera [28], although anecdotal evidence suggests that *E*. *rossii*, a monocalypt species, may be an important food source for koalas in the study region (personal communication with J. Fitzgerald). Regardless, sodium can aid the detoxification and excretion of some plant secondary metabolites (PSMs). For example, plasma sodium concentrations decreased in two species of marsupials (*Trichosurus vulpecula* and *T*. *caninus)* when they were fed tannic acid [29]. Likewise, Freeland et al. [3] provided supplementary sodium to mice on diets containing plant tannins and found a reduction in the degree of erosion of the intestinal mucosa, while snowshoe hares (*Lepus americanus*) ate more of a diet containing PSMs when they had access to sodium-rich soil [30]. It is possible that sodium may aid koalas to ingest and/or process PSMs in *Eucalyptus* leaves. It is also conceivable that this effect may not be consistent between eucalypt subgenera, because they differ in their production of two major classes of PSMs; symphyomyrtles produce formylated phloroglucinol compounds, while monocalypts produce unsubstituted B-ring flavanones [31, 32]. This could be investigated in the future using controlled feeding studies, but has implications for all herbivores ingesting PSM-rich plants in sodium-poor landscapes.

Drainage patterns may help explain why salty barked trees were more likely to occur on flat, rather than sloping landscapes. Studies by William et al [33] of the geomorphology of the Monaro identified several highly saline small lakes as well as other bodies of water that contained salt. These patterns were explained by local drainage features. Although there are no above ground saline water bodies in the study area, it is possible that ancient drainage patterns have left underground seams of highly saline water or a residue of salt-rich deposits that some trees might encounter through their root systems. Trees that access water from such deposits may take up the sodium and export it to the bark. The clustering of salty-barked *E*. *mannifera* trees may be a result of those trees accessing the same below-ground sodium source, and needing to excrete the excess sodium through their bark. Notably, we did not identify any relationship between the underlying geology provided in the Geoscience Australia database [16] and bark sodium from *E*. *mannifera* trees measured in this study. A future study is needed to look at fine scale soil chemistry of the area, and potentially at depth, to determine if this is a key factor that affects sodium concentrations in *E*. *mannifera* bark.

The clustering of salty-barked trees could also be a result of intraspecific variation in the transport of sodium by the roots of *E*. *mannifera*. The classic example of this is in rice, where the sodium content of plants is both highly variable amongst individuals and is also heritable [34]. The reasons are complex, but may be related to transpiration by-pass flow in the roots, which is dependent upon anatomy and development [35]. There is also evidence of significant intraspecific variation in salt tolerance in rice [36]. *Eucalyptus melliodora* trees show strong spatial genetic autocorrelation over short distances, consistent with limited pollen and seed dispersal [37]. *E*. *mannifera* has a similar breeding system, and, therefore, inherited traits that result in higher bark salt concentrations might result in spatially clustered salty-barked trees.

The finding that chewed trees were also clustered may be related to the clustering of salty barked trees in general, but it also appears that koalas chew small amounts of bark from many trees in an area as they search for salty-barked trees. A large number of the chewed trees had test or light chews (66.8% of all chewed trees). Chewed trees may also be clustered because the koala population of the Monaro is patchily distributed across the area [38], resulting in patches of chewed trees where there are more koalas. It is not clear why there was a significant relationship between eastern aspect of slope and an increased occurrence of chewed trees but not salty barked trees per se. It is possible that koalas prefer to spend time on eastern aspects due to the warming morning sun and shelter from afternoon heat, but this has not been reported in other studies.

Although we found a much larger number of chewed *E*. *mannifera* trees in the study area than expected based on the number from previous, but less comprehensive surveys, it is important to note that these included 119 test-chew and 116 lightly chewed trees, which may have lower sodium concentrations than heavily chewed trees. There were not enough trees sampled from each category of chew to comprehensively address this question. However, based on the number of salty-barked trees that were identified through the large-scale random survey of unchewed trees, we can extrapolate that at least 11% of trees in this area have bark sodium concentrations of ≥ 300 mg.kg^-1^ DM, which is based on the amount of sodium in bark with at least moderate levels of chewing.

## Conclusions

Salty-barked trees are not as rare as previously thought, and the identification of salty-barked trees that have not been chewed show that koalas in the Monaro region have not yet fully exploited this resource. However, questions still remain about the mechanism responsible for the salty-bark, and whether there is recruitment for new salty-barked trees in the environment. This information is important for understanding whether salty-barked trees are a renewable or finite resource, since there appears to be a high mortality rate for heavily chewed trees as a result of damage sustained from chewing [7]. We also do not know how frequently koalas visit salty-barked trees, the timing of visits (e.g. season), or the number of trees required in a given home range. There is some evidence to suggest that trees are chewed more often in spring [7], which may correspond to increased sodium requirements during pregnancy and lactation [2]. Understanding how and when resources are used in addition to the overall availability of a resource is essential to determine whether there is sufficient resource availability for a particular population [39].

Animals are often able to exploit resources that are not typical of their normal diet to obtain essential nutrients or micronutrients; however, this does not necessarily ameliorate nutritional deficiencies or their impacts on individuals and populations [1, 4]. Further research is required to investigate the physiology of koalas in the Monaro region to determine whether they are able to obtain sufficient sodium from bark chewing. Notably, koala densities in this area are considered to be very low with an estimated 0.005 animals/ha [38]. Although there is very little information on population-level impacts of sodium poor diets in free-ranging animals, work on captive animals demonstrates that sodium deficiency can have substantial impacts on reproductive success and survival [2]. We propose that sodium deficiency could be a significant barrier to the success of upward migration into high elevation landscapes in response to climate change. Given that many species are predicted to move to higher elevations as a consequence of global warming [11], we need to have a better understanding of the nutritional landscape across a range of elevations, how long it takes animals to identify novel nutritional resources, and how these issues may impact animal populations now and in the future.

## Supporting information

S1 Data The dataset underlying the results described in this manuscript. (XLSX)
